# Effects of amendments on heavy metal immobilization and uptake by *Rhizoma chuanxiong* on copper and cadmium contaminated soil

**DOI:** 10.1098/rsos.181138

**Published:** 2018-08-29

**Authors:** Yanluo Xie, Kemeng Xiao, Yang Sun, Yufeng Gao, Han Yang, Heng Xu

**Affiliations:** Key Laboratory of Bio-Resource and Eco-Environment of Ministry of Education, College of Life Sciences, Sichuan University, Chengdu 610065, Sichuan, People's Republic of China

**Keywords:** amendments, *Rhizoma chuanxiong*, Cd–Cu contaminated soil, accumulation

## Abstract

An improved method was applied for remediating cadmium and copper co-contaminated soil and reducing the metal concentration in *Rhizoma chuanxiong*. Pot experiments were conducted with six amendments (composed with bentonite, phosphate, humic acid, biochar, sepiolite powder, etc.). The results showed that soil pH, biological activities (soil enzymatic activities and microbial counts) and *R. chuanxiong* biomass were greatly improved with the addition of amendments in all treatments, especially in T3 and T6. Also, amendments effectively decreased the concentration of malondialdehyde and H_2_O_2_ in *R. chuanxiong*. In the T3 treatment, the bio-available Cd and Cu in soil were significantly decreased by 0.53 and 0.41 mg kg^−1^, respectively. Meanwhile, the amendment in T3 reduced Cd and Cu accumulation in *R. chuanxiong* about 45.83 and 39.37%, respectively, compared to T0. Moreover, the Fourier transform infrared spectroscopy spectra showed the surface functional groups of every amendment. To conclude, this study offers an effective and environmental method to reduce metal accumulation in *R. chuanxiong* on heavy metal co-contaminated soil.

## 1. Introduction

With the development of industrialization, urbanization and construction, heavy metals have generated several serious environmental problems. Heavy metals such as aluminium (Al), copper (Cu), lead (Pb), cadmium (Cd) and chromium (Cr) are easy to transfer into soil and water, which will pose extreme toxicity to plants and aquatic organisms [1,2]. For example, Cd absorbed by plant through rhizospheres will inhibit reactive oxygen species (ROS)-detoxifying enzymes, thus affecting plant growth [3]. The high concentration of heavy metals will seriously threaten humans' health through the food chain [4,5]. Xu *et al.* [6] found that people at high risk of HIV had higher blood concentrations of metals including Cd, Pb and mercury (Hg) compared to those with low risk of HIV. Besides, Cu will lead to the generation of ROS that will damage cellular constituents, reducing growth and yield of plants [3]. Moreover, combined pollution with various metals has become the main form of heavy metal pollution in soil. Therefore, the study of remediating heavy metal-contaminated soil attracts more and more attention all over the world.

*Rhizoma chuanxiong* (*Ligusticum chuanxiong Hort*, Umbelliferae), the dried rhizome of the Umbelliferae plant species *R. chuanxiong Ligusticum chuanxiong* Hort, is one of the most popular and oldest herbal medicines in the world [7]. *Rhizoma chuanxiong* has been used for thousands years in traditional Chinese, Japanese and Korean folk medicine [8], and applied to the prescriptions of angina pectoris [9], hypertension [10], ischaemic stroke [11], etc*.* In addition, *R. chuanxiong* has attracted many researchers' attention owing to the ability of accumulating heavy metals. For example, Li *et al.* [12] found that the accumulation capacity of *R. chuanxiong* to Cd and Cu was more remarkable, especially to Cd, compared to Pb and Hg. Up to now, there are only few reports about the methods of reducing metal uptake by *R. chuanxiong.* Therefore, to ensure the safe use of *R. chuanxiong*, it is of key significance to reduce heavy metal concentration in *R. chuanxiong*.

Many methods have been applied to reduce the availability of heavy metal in soil and plants, including physical, chemical and biological methods. Chemical immobilization is to decrease the concentration of dissolved contaminants by sorption [13], which will decrease metal availability to plants [14]. Soil amendments, such as organic matter, and phosphate, can reduce the solubility of metals in soil [14]. The method in this experiment was to add amendments into soils, which not only provided nutrition for plants, but also reduced the bioavailability of heavy metals.

Bentonite clays is a cost-effective amendment for stabilization of heavy metals in soil owing to its isomorphic substitution, permanent negative charges at surfaces as well as its environmental compatibility and ready availability [15–17]. Phosphate (i.e. KH_2_PO_4_, CaH_2_PO_4_ and H_3_PO_4_), one of the most common amendments [18], could reduce the availability of heavy metals and provide nutrition for crops [19]. Hence, bentonite and phosphate are preferable materials to immobilize heavy metals and promote plant growth. Biochar is introduced as soil amendments to stabilize or passivate heavy metals in the contaminated soils and improve quality of polluted soil [20]. Humic acid has both directly or indirectly positive effects on plant growth and health [21]. Silicon fertilizer has a lot of advantages for plant growth in metal-contaminated soils such as reducing metal uptake, increasing root growth biomass and so on [22]. Biological matrix can improve utilization efficiency of organic carbon and promote immobilization of organic carbon [23].

The main objectives of this experiment were to: (i) investigate reduction in metal concentration in *R. chuanxiong* by applying different amendments; (ii) evaluate remediation efficiency of amendments by estimating malondialdehyde (MDA), hydrogen peroxide (H_2_O_2_) and metal accumulation in *R. chuanxiong* coupled with the variety of soil quality (pH, soil enzymatic activity and respiration, microbial counts and metals bioavailability); and (iii) explore the correlation between soil pH, the content of available heavy metals in soil and accumulation of heavy metals in *R. chuanxiong*.

## 2. Material and methods

### 2.1. Pot experiments

In this study, soils were collected from the campus of Sichuan University, Chengdu, China, where a remotely experimental site has been applied for scientific research about heavy metals for many years. The collected soil samples were air-dried and passed through a 2 mm sieve, then homogenized adequately for uniform distribution of heavy metals and other substrates. The soil basic prosperities are given in table 1, and the concentrations of Cd and Cu in soils were about 8.9 and 164.2 mg kg^−1^, relatively. The components of amendments were bought from Tianjing Environmental Protection Monitoring Station, China.

**Table 1.** Basic properties of the soil in this experiment.

properties	values
pH	6.5
cation exchange capacity (CEC, cmol kg^−1^)	12.1
sand (0.006–2 mm) (%)	68.1
clay (less than 0.002 mm) (%)	6.2
silt (0.0002–0.06 mm) (%)	25.7
organic content (g kg^−1^)	16.8
Cd (mg kg^−1^)	8.9
Cu (mg kg^−1^)	164.2

Plastic pots (height 13 cm and diameter 20 cm) were used for this experiment. After air-drying, soil samples (2 kg) were mixed with 1.5% of amendments (w/w). The amendments mostly consisted of bentonite and phosphate, and the experiment set seven experimental groups with three replicates, including six groups (T1–T6) added amendments and a group with (T0) non-addition as a control (table 2). Two plants of *R. chuanxiong* (bought from Sichuan Academy of Agricultural Sciences) were planted in each pot, then cultivated from August to November with an average temperature of 25°C indoors.

**Table 2.** Composition and proportion of amendments in T1, T2, T3, T4, T5 and T6.

treatment	composition	proportion
T1	bentonite : phosphate : humic acid : biochar	58% : 2% : 2% : 38%
T2	bentonite : phosphate : biochar : silicon fertilizer	85% : 3% : 7% : 5%
T3	bentonite : phosphate : biological matrix : compost	40% : 17% : 26% : 17%
T4	bentonite : phosphate : biological matrix : silicon fertilizer	40% : 15% : 25% : 20%
T5	bentonite : phosphate : humic acid : biochar	50% : 15% : 15% : 20%
T6	sepiolite powder : biochar : dolomite powder : KH_2_PO_4_	40% : 30% : 20% : 10%

Approximately three months later, *R. chuanxiong* were harvested successively and washed with deionized water, then wet weights were recorded. *Rhizoma chuanxiong* were dried at 60°C for 3 days in an oven.

### 2.2. pH of soils

One gram of air-dried soil was weighed in a 5 ml breaker, and added to 2.5 ml of deionized water without CO_2_ [24]. The solution was stood for 30 min and measured by pH meter (SevenCompact-s210).

### 2.3. Soil biological activities

Soil samples were collected from each pot after *R. chuanxiong* was harvested to estimate soil enzyme activities and bacteria counts. The measurement of dehydrogenase was through a spectrophotometer as described by Zhou *et al.* [25] with minor modifications by prolonging the reaction time to 48 h. Activities of fluorescein diacetate (FDA) hydrolysis, urease, acid phosphatase and invertase were assayed by the methods of Adam & Duncan [26], Yan [27], Alef & Nannipieri [28] and Gu [29], respectively. Dehydrogenase activity was determined spectrophotometrically at 492 nm and expressed as microgram triphenylformazan (TPF) per soil per hour. FDA activity was determined spectrophotometrically at 490 nm and presented as the content of fluorecien in dry soil (µg g^−1^). The activity of urease was spectrophotometrically determined at 578 nm and presented as mg NH_4_^+^ g^−1^ soil. The activity of invertase was determined spectrophotometrically at 508 nm and presented as milligram glucose per gram soil per 24 h. Bacterial counts in soil were counted on LB agar medium through the spread-plate method as described by Liu *et al.* [30].

### 2.4. Heavy metals analysis

Heavy metals in *R. chuanxiong* and soil samples was detected by atomic absorption spectroscopy (VARIAN, SpecterAA-220Fs) as described by Wu *et al.* [31]. Dried powdered and sieved samples of 0.2 g soil and plant were digested by HCLO_4_ (5 : 4 : 3, v/v/v).

The soil samples were collected to measure metal bioavailability by a toxicity characteristic leaching procedure (TCLP) and 0.01 M CaCl_2_ extraction [32,33]. Two grams of air-dried sieved (2 mm) soil were added to 40 ml of 0.11 mol l^−1^ HAc at pH 2.88 ± 0.05 and shaken for 18 h. Then the solution was filtered with a 0.45 µm filter membrane for Cd and Cu analysis. At room temperature (about 25°C), 2.5 g of air-dried soil samples were incubated for 3 h in a shaker at 250 rpm with 12.5 ml of 0.1 mol l^−1^ CaCl_2_. Then the solution was centrifuged at 3000 rpm for 10 min and filtered with a 0.45 µm filter membrane. All the extraction was determined by flame atomic absorption spectrometry.

### 2.5. Malondialdehyde determination

According to the method of Liu *et al.* [30] with some modifications, *R. chuanxiong* tissues (1 g) were ground with 2 ml of 10% trichloroacetic acid (TCA) and quartz sand, then homogenized with 8 ml of 10% TCA. The absorbance was determined at 450, 532 and 600 nm. MDA was quantified with equation (2.1):

2.1 $$\text{MDA concentration}\;(\mu \mathrm{mol}\;{\text{l}}^{-1})=6.45\times (\text{OD532}-\text{OD600})-0.56\times \text{OD450}.$$

### 2.6. H_2_O_2_ determination

H_2_O_2_ determination was conducted following the steps of the hydrogen peroxide reagent kit from Nanjing Jiancheng Bioengineering Institute, China, and the absorbance was measured at 450 nm with spectrophotometry.

### 2.7. Fourier transform infrared spectra amendment assay

The Fourier transform infrared spectroscopy (FTIR) spectra of amendments prepared as KBr discs were recorded in a Perkin-Elmer Spectrum 100 Model Infrared Spectrophotometer to examine functional groups of amendments [34]. FTIR spectra were recorded in the range of 400–4000 cm^−1^ at a resolution of 2 cm^−1^.

### 2.8. Data analysis

The mean and standard deviation of the three replicates were calculated in this study. Statistical significance was performed through using one-way ANOVA in SPSS 17.0, and the mean values were compared using the least significant difference calculated at a significant level of *p* < 0.05. All figures were performed by using Origin 8.5 software. Also, SPSS version 21.0 for Win was used to reflect the multivariate linear relationship among contaminated soil physicochemical characteristic, microbial abundance and metal fractions.

## 3. Results and discussion

### 3.1. Soil pH change

**Figure 1.**
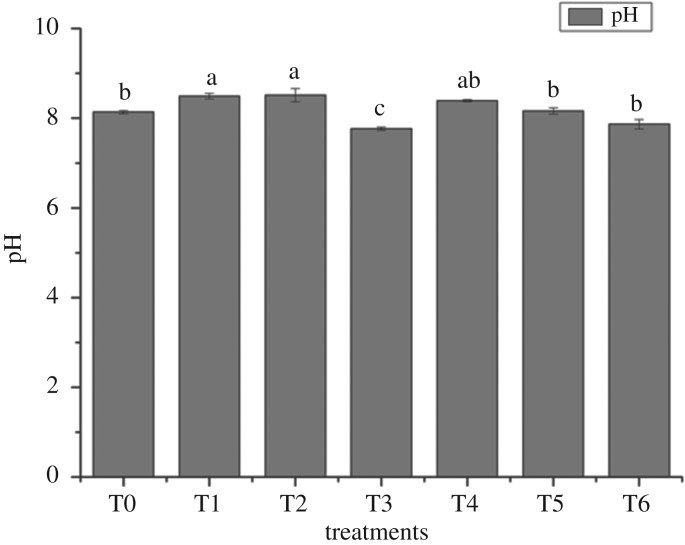
Effects of soil pH with different amendments. pH of soils with different treatments after *R. chuanxiong* was harvested. Error bar represents the standard deviation of three samples. Columns denoted by different lowercase letters (a, b, c) indicate significant (*p* < 0.05) differences among different treatments. T0, treatment without amendment; T1–T6, treatments with different amendments.

The pH values of soil with different treatments at the end of the experiment are shown in figure 1. The soil pH increased compared to the initial pH (pH = 7.93), except for T3 (pH = 7.77) and T6 (pH = 7.87), but there were no significant differences (*p* > 0.05) among T0, T3 and T6. T2 presented the highest increase in soil pH. Bentonite which was the same component in all amendments had a positive effect on soil pH [35], therefore causing the slight increase in soil pH in this experiment.

### 3.2. Analysis of metal availability

**Figure 2.**
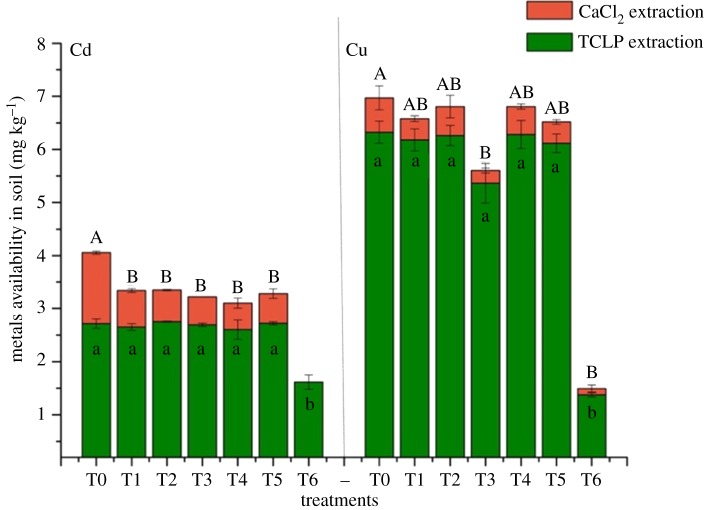
Effects of heavy metal availability in soil. Available metals in soil with different treatments after *R. chuanxiong* was harvested. Error bar represents the standard deviation of three samples. Columns denoted by different lowercase letters (a, b, c, d) and capital letter (A, B) indicate significant (*p* < 0.05) differences among different treatments. T0, treatment without amendment; T1–T6, treatments with different amendments.

TCLP is always used for heavy metal leached fraction concept [33] and CaCl_2_ extraction can remove metals by ion exchange with Ca^2+^ and/or complexation with the chloride species [32]. In this experiment, bio-available metals were extracted by TCLP and CaCl_2_ extraction methods. It could be observed that the available metals (Cu, Cd) decreased compared with T0, especially for Cd extraction by CaCl_2_ (figure 2). For example, CaCl_2_-extracted Cd in T6 was too small to detect. The CaCl_2_-extracted Cd in T3 and T4 both decreased from around 1.34 to 0.5 mg kg^−1^ and showed a significant decrease of about 0.84 mg kg^−1^, compared to other treatments. The order of CaCl_2_ extracting Cd in soils was T4 < T3 < T5 < T1 < T2, T6 as exceptional data. In the T3 treatment, the immobilization effect on Cu was also excellent with the decrease rate of 78.67% in CaCl_2_-extracted Cu. The order of CaCl_2_ extractable Cu from soils was T6 < T3 < T1 < T5 < T4 < T2, while the order of TCLP extracting Cu was T6 < T3 < T5 < T1 < T2 < T4. Above all, with the least content of available heavy metals, T6 showed the best immobilized effect on heavy metals.

Many studies have documented a negative correlation between soil pH and heavy metal availability [36]. The increase in pH in this experiment can benefit precipitation and sorption of heavy metals. Besides, some compositions of amendments, such as bentonite [37], phosphate [38], had strong adsorption capacity towards heavy metals, consequently reducing the toxicity of heavy metals.

### 3.3. Microbial counts and respiration intensity of soil

**Figure 3.**
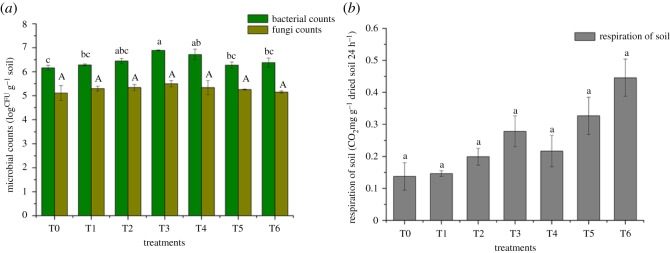
Effects of microbial activities in soil. Microbial counts including bacterial and fungi (*a*) and soil respiration (*b*) from different treatments. Error bar represents the standard deviation of three samples. Columns denoted by different lowercase letters (a, b, c) in bacterial counts and capital letter (A) in fungi counts indicate significant (*p* < 0.05) differences among different treatments. T0, treatment without amendment; T1–T6, treatments with different amendments.

The microbial counts including bacteria and fungi counts and soil respiration are shown in figure 3*a*. It could be observed that bacterial counts had a slight increase in all treatments compared to T0 (log6.12 CFU g^−1^ soil). Bacterial counts in T3 (log6.89 CFU g^−1^ soil) increased most, followed by T4 (log 6.72 CFU g^−1^ soil) compared to T0. Also, the highest and lowest fungi number were presented in T3 (log 5.50 CFU g^−1^ soil) and T0 (log5.11 CFU g^−1^ soil), respectively. On the whole, the number of microbes increased after the immobilization process, indicating that the addition of amendments contributed to soil micro-environment improvement.

The respiration intensity of soil is shown in figure 3*b*. It was clear that the respiration intensity in T6 (0.45 CO_2_mg g^−1^ dried soil 24 h^−1^) was around three times that of T0 (0.14 CO_2_mg g^−1^ dried soil 24 h^−1^); meanwhile, the respiration intensity of T3 (0.28 CO_2_mg g^−1^ dried soil 24 h^−1^) and T4 (0.22 CO_2_mg g^−1^ dried soil 24 h^−1^) were both almost two times more than in T0. Besides, the respiration intensity in other treatments also increased more or less.

The available heavy metals decreased, which reduced the environmental stress to microbes. Thus, the microbial counts and respiration intensity increased. Soil bacterial and fungal population were significantly and positively correlated with soil organic matter [39]. Amendments in this experiment provided amount of organic matter that could be used by microbes, which might be a reason for the microbial counts increase. For example, biological matrix (mainly straw powder), one of the same components of amendments in T3 and T4, could motivate soil microbial activity owing to the richness of N, P, K and trace elements [40]. Besides, other components also have a positive effect on microbial activity. For example, biochar in T1, T2, T5 and T6 could improve soil aeration through enhancing oxygen (O_2_) diffusion, thereby stimulating aerobic activity in soil [41].

### 3.4. Soil enzymatic activities

**Figure 4.**
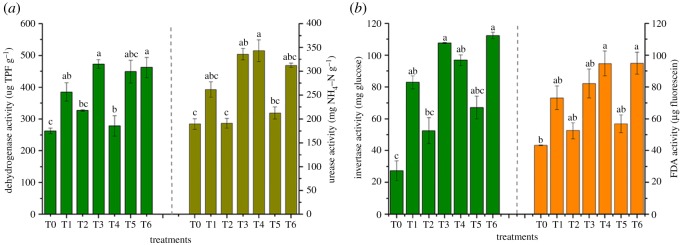
Effects of soil enzymatic activities. Enzymatic activities of soils with different treatments after *R. chuanxiong* was harvested. (*a*) Dehydrogenase activity, urease activity; (*b*) invertase activity, FDA activity. Error bar represents the standard deviation of three samples. Columns denoted by different lowercase letters (a, b, c) indicate significant (*p* < 0.05) differences among different treatments. T0, treatment without amendment; T1–T6, treatments with different amendments.

The activity of dehydrogenase in soil is shown in figure 4*a*. Dehydrogenase activity showed different degrees of increase in all treatments compared to T0 (262.46 mg TPF g^−1^). It indicated that a light fluctuation happened in T2 (326.69 mg TPF g^−1^) and T4 (278.65 mg TPF g^−1^). Also, T3 and T6 presented the largest activities of dehydrogenase, about 472.86 and 461.90 mg TPF g^−1^, respectively.

For urease activity, there was no significant difference (*p* < 0.05) among all treatments with comparison to T0. But higher activity presented in T3, T4 and T6 (all above 300 mg NH_4_–N g^−1^) compared to T2 (just about 190 mg NH_4_–N g^−1^) (figure 4*a*).

Invertase activity is by far less sensitive to heavy metals than dehydrogenase and urease [42]. Yet, it was observed that a significant change arose in this experiment (figure 4*b*). The highest activity of invertase presented in T3 and T6, in specifically, about 108 and 112 mg glucose g^−1^, respectively, both of which were four times than that of T0 (27 mg glucose g^−1^). Compared to T0, invertase activity increased to approximately 50 glucose g^−1^ in T1, and nearly 70 mg glucose g^−1^ in T4. The activities of invertase in T2 and T5 increased slightly, compared to the control.

The activity of FDA presented increased trends with the addition of amendments compared to T0 (figure 4*b*). More concretely, higher FDA activity presented in T3, T4 and T6 treatments, which were around two times greater than the activity in T0. Besides, the FDA activity showed a slight increase in T1, T2 and T5 in comparison to the control.

The increase in soil enzymatic activities cooperated with the increase in soil microbes [39,43]. With the reduction in heavy metal toxicity, microbial activity increased as a result of enzymatic activity increasing. Besides, the addition of amendments improved soil physicochemical properties, such as high cationic exchange capacity of bentonite [44], and phosphate increasing extractable soil inorganic P [45]. Above all, it was obvious that amendments in T3, T4 and T6 showed a more positive effect on enzymatic activity.

### 3.5. Biomass of *Rhizoma chuanxiong*

**Figure 5.**
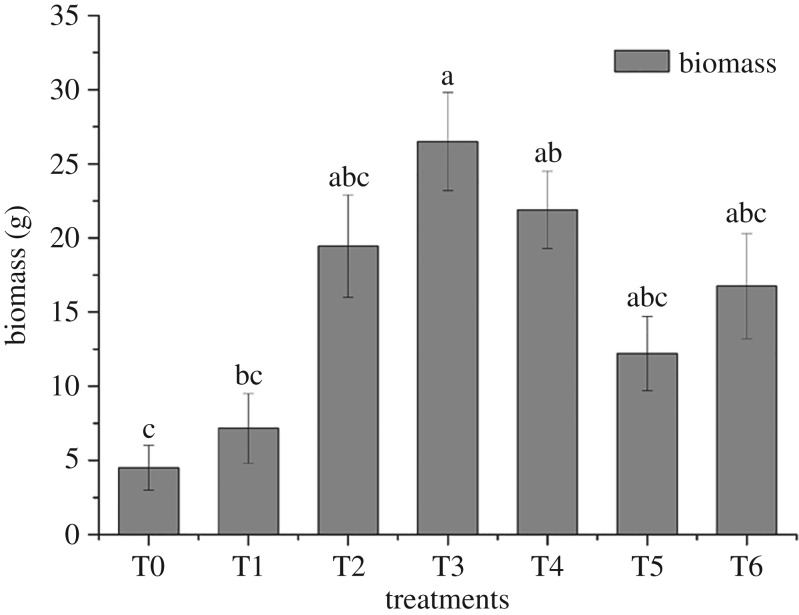
Effects of *R. chuanxiong* biomass. Biomass of *R. chuanxiong* in different treatments. Error bar represents the standard deviation of three samples. Columns denoted by different lowercase letters (a, b, c) indicate significant (*p* < 0.05) differences among different treatments. T0, treatment without amendment; T1–T6, treatments with different amendments.

To evaluate the effect of amendments on the growth of *R. chuanxiong* in metal-contaminated soil, biomass of *R. chuanxiong* was recorded (figure 5). Compared to T0, the growth of *R. chuanxiong* was promoted in every treatment. Moreover, the biomass of *R. chuanxiong* in T3 was five times that of T0, and the growth in T1 was the least among treatments. These results indicated amendments played a positive role in the growth of *R. chuanxiong* in polluted soil.

The amendment provided nutrition for plants and adjusted soil pH, which could be propitious to *R. chuanxiong* growth. Meanwhile, plant biomass increase, in turn, can dilute the account of heavy metals with a ‘dilution effect’ [46]. The results in this experiment indicated that application of amendments could promote *R. chuanxiong* growth.

### 3.6. Heavy metal concentrations in *Rhizoma chuanxiong*

**Figure 6.**
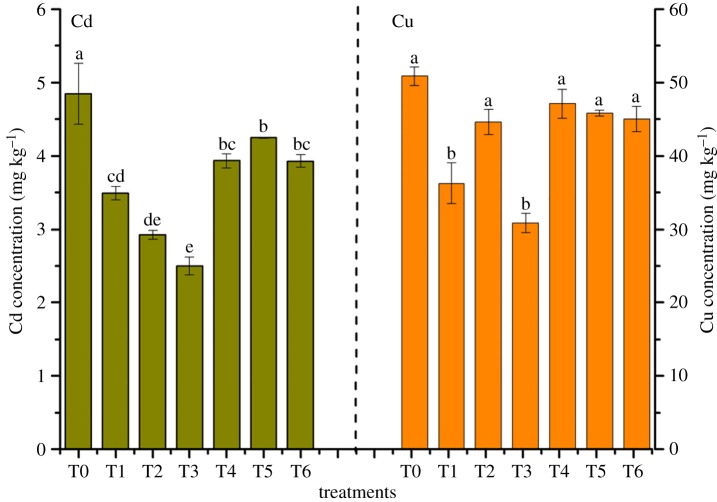
Effects of heavy metal concentration in *R. chuanxiong*. Metal concentrations with different treatments after *R. chuanxiong* was harvested. Error bar represents the standard deviation of three samples. Columns denoted by different lowercase letters (a, b, c, d, e) indicate significant (*p* < 0.05) differences among different treatments. T0, treatment without amendment; T1–T6, treatments with different amendments.

The concentrations of Cd and Cu in *R. chuanxiong* are presented in figure 6. On the whole, metals declined in *R. chuanxiong* with the addition of amendments compared to T0. Especially in the T3 treatment, the contents of Cd and Cu showed the remarkable declining rates by 45.83% and 39.37%, respectively. It was interesting that metal uptake by plants had a different tendency as enzymatic activity and microbial biomass. The difference between T3 and T6 on metal availability in soil and metal concentration in plants might indicate that the amendment in T3 had a more positive influence on reduction in metal bioavailability in soil and uptake by plants. Besides, although the amendment of T6 could immobilize metals, it had little effect on preventing plants uptake in this study. The difference of metal availability in soil and plant between T3 and T6 could be explained as follows. It could be observed that the bentonite in T3 and sepiolite powder in T6 had the same proportion of amendments, about 40%. The sepiolite powder had greater specific surface area and greater ion exchange capacity, while montmorillonite as the main component of the bentonite had low crystallinity values [47], which made the sepiolite powder have a stronger adsorption capacity to heavy metals. Thus, the available heavy metals in T6 were less than those in T3. However, the sepiolite powder was rich in Mg^2+^ and Ca^2+^ [48] whose concentrations in soil had a positive relation to the accumulation of heavy metal in plants. Therefore, the concentrations of Cd and Cu in *R. chuanxiong* in T3 were lower than those in T6.

The phenomena about the reduction in heavy metal concentrations in *R. chuanxiong* might be explained as follows: (i) the reduction in available heavy metals made it difficult for metals to enter the plant; (ii) amendments used in this experiment were full of organic matter which played an important role in retaining soil Cd and in decreasing its availability to plants through cation exchange capacity (CEC) [49]. As Marchand *et al*. researched [50], soil organic matter is pivotal for controlling metal (e.g. Fe, Cu and Pb) bioavailability from strong organometallic complexes. For example, in the research of Sun *et al*. [51], bentonite can reduce metal exchangeable fraction, in alkaline soils, resulting the decrease in metal bioavailability; (iii) some research indicated that phosphate could reduce metal dissolution and transport from contaminated soil by increasing the efficiency of metal-phosphate mineral formation, then precipitating [52]; and (iv) the better effect on decreasing metal availability in soil and uptake by *R. chuanxiong* in T3 might indicate the biological matrix and compost was better for bentonite and phosphate on immobilizing metals.

### 3.7. Antioxidant stress of *Rhizoma chuanxiong*

H_2_O_2_ is one of the ROS molecules which is formed upon incomplete reduction in oxygen [53]. The overproduction of H_2_O_2_ may lead to cell death and oxidative damage [54]. MDA is produced from cell membrane peroxidation [55]. The metals can increase the concentration of H_2_O_2_ and MDA in plants [56]. Thus, the production of H_2_O_2_ and MDA can indicate metal stress on plants [57].

**Figure 7.**
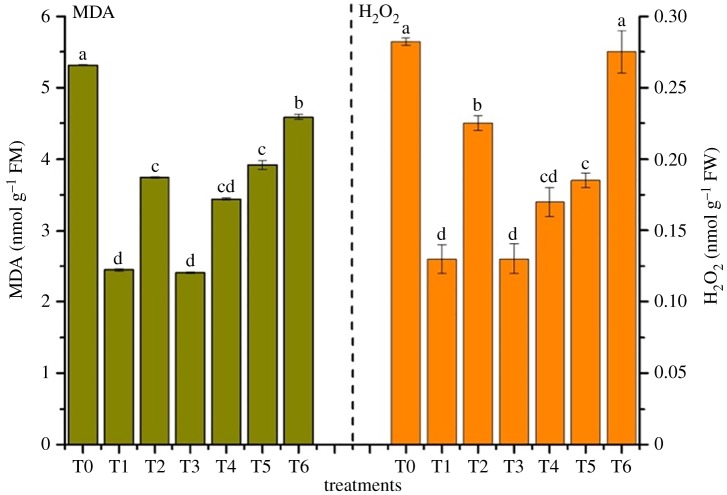
Effects of antioxidant system in *R. chuanxiong*. MDA and H_2_O_2_ concentration of *R. chuanxiong* in different treatments. Error bar represents the standard deviation of three samples. Columns denoted by different lowercase letters (a, b, c, d) indicate significant (*p* < 0.05) differences among different treatments. T0, treatment without amendment; T1–T6, treatments with different amendments.

The concentrations of H_2_O_2_ and MDA are shown in figure 7. Compared to T0, there was a decreased tendency but no significant difference on H_2_O_2_ and MDA with the application of amendments among all treatments. MDA decreased by 12.83–54.34% and H_2_O_2_ decreased by 13.33–57.54% in all treatments. It proved that all treatments presented positive effects on reduction in H_2_O_2_ and MDA levels in *R. chuanxiong*, compared to T0.

Environmental stress, such as salinity, temperature extremes and high metal contamination, could decrease the activities of superoxide dismutase (SOD) and increase the activities of catalase (CAT) and peroxidase (POD) [58–60]. Because SOD disproportionates O_2_^−^ to produce H_2_O_2_, H_2_O_2_ could be decomposed to H_2_O and O_2_ catalysed by CAT [57]. So, the amendment decreased the contents of available metals in soil, thus reducing the toxicity of heavy metals to plants. Then, the environmental stress on plants reduces, thereby reducing the values of H_2_O_2_ and MDA in *R. chuanxiong*.

### 3.8. The Fourier transform infrared spectra amendments

**Figure 8.**
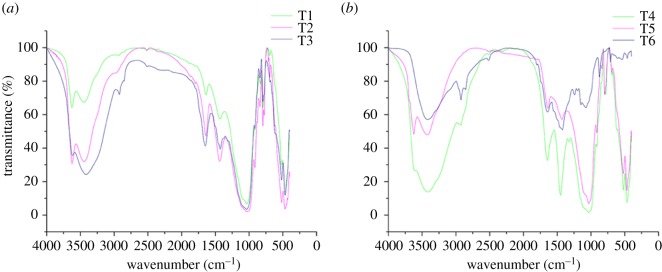
FTIR of all amendments in different treatments.

The results of FTIR are shown in figure 8. The absorption band that appeared at 3620 cm^−1^ might detect the inner hydroxyl groups [61], and it was not observed in T6. Besides, the absorption of 2856 cm^−1^ are ascribed to stretching vibration of methylene (−CH_2_) groups in antisymmetric and asymmetric [62], which are mainly obvious in T1, T3 and T6. The sorption bands at 1499 and 1465 cm^−1^ expressed the C–H bend from CH^2^ and C–O bend from carboxylate ions, and the P = O from phosphate, C–O–P and P–O–P were absorbed at 1241, 1197 and 1148 cm^−1^ [63–65]. The bands below 500 cm^−1^ represent the C_a_ = C_a_ torsion, C–OH_3_ torsion of the methoxy group and ring torsion of phenyl [66]. The vibrations at near 3620, 2856, 1465, 1128 and 1035 cm^−1^ might indicate that –OH, –NH^2^, –CH^2^ and phosphate were related to the immobilization and complexation progress of Cd and Cu. Moreover, compared to T6, the vibration of 1241, 1197, 1148, 1074 cm^−1^ and below 500 cm^−1^ might imply that P=O, C–O–P, P–O–P, the C_a_=C_a_ torsion, C–OH_3_ torsion of the methoxy group and ring torsion of phenyl were involved in metals (Cd, Cu) uptake by *R. chuanxiong*.

## 4. Conclusion

In the present study, the addition of amendments reduced the bioavailability of heavy metals and concentration in *R. chuanxiong*. Also, the amendments slightly improved soil pH, and soil biological activities (enzymatic activities, microbial counts). In all kinds of treatments, T3 and T6 showed a better effect on reducing metal availability in soils, which indicated that the recipes of amendment in T3 and T6 are more suitable to immobilizing Cd and Cu, compared to other treatments; whereas, amendment of T1 and T3 appeared to work better to reduce metal concentration in *R. chuanxiong*. In summary, results from this experiment declared that the amendment in T3 was the best treatment to reduce soil metal toxicity and concentration in *R. chuanxiong*. This experiment provided an effective and environmentally friendly pathway to remediate heavy metal-contaminated soil *in situ*.

## Data accessibility

This article does not contain any additional data.
